# Application of cardiovascular 3-dimensional printing in Transcatheter aortic valve replacement

**DOI:** 10.1186/s13619-022-00129-4

**Published:** 2022-09-19

**Authors:** Yanyan Ma, Yu Mao, Guangyu Zhu, Jian Yang

**Affiliations:** 1 Department of Cardiovascular Surgery, Xijing Hospital, Air Force Medical University, 127 Changle West Road, Xi’an, 710032 Shaanxi China; 2 School of Energy and Power Engineering, Xi’an Jiaotong University, Xi’an, China

**Keywords:** 3D printing, Transcatheter aortic valve replacement, Models, Aortic root

## Abstract

Transcatheter aortic valve replacement (TAVR) has been performed for nearly 20 years, with reliable safety and efficacy in moderate- to high-risk patients with aortic stenosis or regurgitation, with the advantage of less trauma and better prognosis than traditional open surgery. However, because surgeons have not been able to obtain a full view of the aortic root, 3-dimensional printing has been used to reconstruct the aortic root so that they could clearly and intuitively understand the specific anatomical structure. In addition, the 3D printed model has been used for the in vitro simulation of the planned procedures to predict the potential complications of TAVR, the goal being to provide guidance to reasonably plan the procedure to achieve the best outcome. Postprocedural 3D printing can be used to understand the depth, shape, and distribution of the stent. Cardiovascular 3D printing has achieved remarkable results in TAVR and has a great potential.

## Background

With the continuous development of digital precision medicine, the requirements for digital modeling are particularly important in cardiovascular diseases (Corrigan 3rd et al. 2019). The emergence of 3D printing has brought new ideas and methods to the diagnosis and treatment of many complex cardiovascular diseases. Doctors could use imaging data to complete 3D reconstructions and models and provide surgeons with a clear understanding of the individualized anatomical structures (Alasnag et al. 2020; Bompotis et al. 2017; Alamir et al. 2020).

Compared with traditional thoracotomy, transcatheter aortic valve replacement (TAVR) results in less trauma and a better prognosis, with reliable safety and efficacy in elderly patients with aortic stenosis or regurgitation (Winter et al. 2020). Traditional thoracotomy differs from TAVR in that, with the former, it is impossible to view the aortic root directly, let alone open the heart to observe the internal anatomical structures (Claessen et al. 2021). In addition, imaging data may only provide a 2-dimensional view, and surgeons still need to study and estimate further before beginning the operation (Sanchez et al. 2019).

The emergence of 3D printing is of great help to those performing TAVR. Studies have found that the preprocedural computed tomography (CT) data for the aortic root can be used for 3D reconstruction and printing because it provides a more intuitive understanding of the anatomical structures (Levin et al. 2020; Zelis et al. 2020). Hosny et al. suggested that the combination of cardiovascular 3D printing and TAVR allows surgeons to view the anatomical structures directly via the 3D printed model of the aortic root (Hosny et al. 2019). Furthermore, Nam et al. displayed the 3D printed model may also improve communication between doctors and patients and between young clinicians and medical students and the physicians and surgeons who train them (Nam et al. 2021). In addition, researchers used the 3D printed model in vitro for the simulation of TAVR in order to assess the potential for coronary obstruction, paravalvular leakage, conduction block, and other serious complications, in order to develop individualized preprocedural plans and to help improve the safety and efficacy (Gardin et al. 2020; Ferrari et al. 2019) of the procedure. Thus, the procedure is greatly simplified and becomes more accurate and effective.

## Methods of 3-dimensional printing

### 3-dimensional printing process

**Fig. 1:**
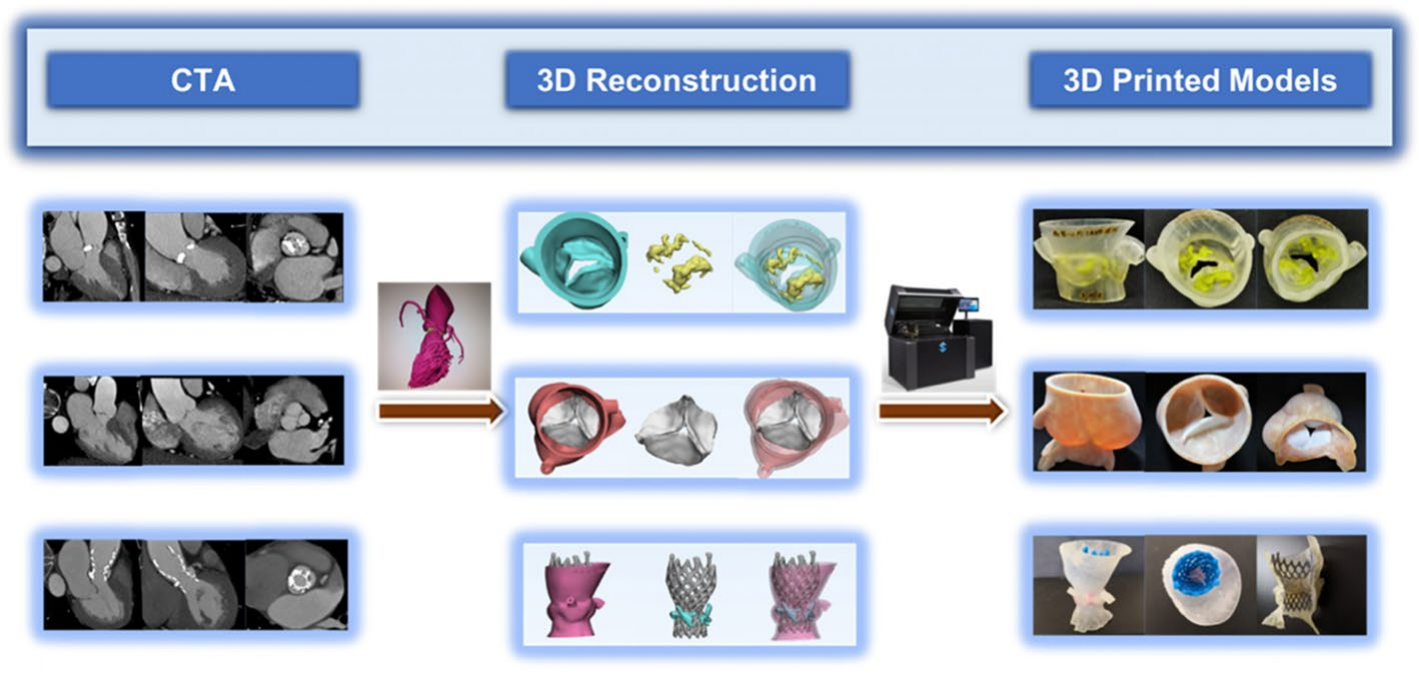
Pre- and postprocedural computed tomography angiography (CTA) data were used for the process of 3-dimensional (3D) printing

Patient CT data in Digital Imaging and Communications in Medicine (DICOM) format are imported into Materialise Mimics version 21.0 software (Materialise, Leuven, Belgium), and three orthogonal sections are established based on the multiplane reconstruction function of the software. The positions of the three orthogonal sections can be adjusted at will. When obtaining the images of the lesion, the aortic root is segmented, and the delineated area can be reconstructed to obtain the initial 3D model of the aortic root. Materialise 3-Matic software (Materialise, Leuven, Belgium) is used to digitally cut, smooth, repair, and extract the shell of the model; the structures of the valve and the distribution of the calcified areas are completely reconstructed. The different parts of the digital model are distinguished by different colors to fully represent the multidimensional structures, such as the morphology, the distribution, and the interface. Finally, the model is exported into Standard Tessellation Language (STL) format. Then the STL files are imported into a Stratasys Polyjet 850 multimaterial full-color 3D printer (Stratasys, Inc., Eden Prairie, MN, USA), and different materials are selected to represent different tissues. Then the models are printed (Greil et al. 2007; Byrne et al. 2016; Halliburton et al. 2012) (Fig. 1). Overall, the printing process includes a series of post-processing procedures (such as removing, grinding, clarifying, and polishing the support structures) to obtain an exquisite, colorful 3D multimaterial model for clinical use.

## Applications of 3-dimensional printing in transcatheter aortic valve replacement

### Visualization of the anatomical structures of the aortic root

**Fig. 2:**
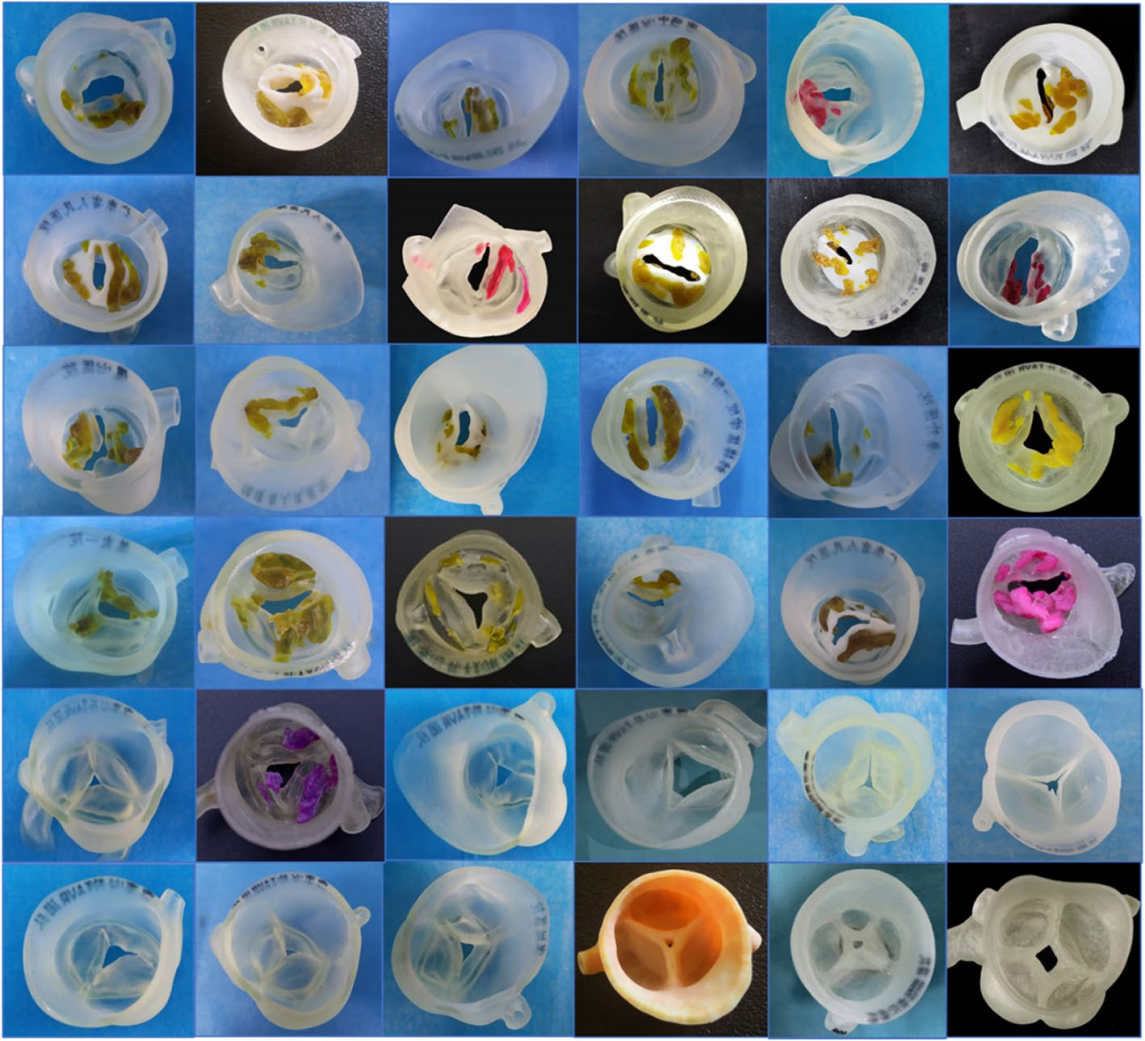
Multicolor and multimaterial 3-dimensional printed models of aortic roots with different types of leaflets (leaflets as soft materials; calcifications as hard materials)

Gallo et al. (Gallo et al. 2016) and Hague and Leipsic (Hague and Leipsic 2012) demonstrated that aortic root models with different leaflet morphologies could be printed and could be helpful in illustrating the anatomical structures. Patient preprocedural CT image data are reconstructed and printed using 3D printing technology. The realistic model can provide the surgeons and the patients with a clear, intuitive understanding of the morphology of the leaflet, aortic valve prolapse with regurgitation, distribution of calcification, the sinus, and the ascending aorta. In addition, doctors use balloon-expandable valves for simulations in vitro and even more carry out simulations with pulsatile flow platforms. When this information is combined with an evaluation of the images, the diagnosis and treatment may be planned more accurately before the TAVR procedure to ensure its success (Fig. 2).

### Preprocedural assessment

As the number of TAVR procedures continues to increase, it is particularly important to prevent common complications. The general postprocedural complications of TAVR include paravalvular leakage, coronary obstruction, vascular complications, arrhythmia, stroke, and acute kidney injury. Maragiannis et al. (Maragiannis et al. 2015) and Ventola (Ventola 2014) demonstrated that using 3D printing in TAVR to evaluate the possibility of complications is a new concept. Schmauss et al. (Schmauss et al. 2012) and Ferrari et al. (Ferrari et al. 2017) showed that the aortic root model was reconstructed by 3D printing for preprocedural planning and simulation.

### Coronary obstruction

Coronary obstruction refers to the phenomenon of myocardial infarction whereby the stent pushes the valve or the calcifications into the coronary artery after TAVR. Akinseye et al. (Akinseye et al. 2018) and Chakraborty et al. (Chakraborty et al. 1995) demonstrated that the main risk factors affecting coronary obstruction probably are as follows: (1) The distance between the coronary opening and the annular plane is too short, especially when the distance is < 12 mm; (2) longer and thickened leaflets; (3) diameter of the Wallis sinus < 30 mm; and (4) calcifications at the margins of the left and right coronary sinuses. Even though the follow-up results of some patients were good after TAVR, delayed coronary obstruction could still occur. As a result, if the type of prosthetic valve is unknown, any complication that occurs within 1 week after surgery needs to be investigated more vigilantly.

**Fig. 3:**
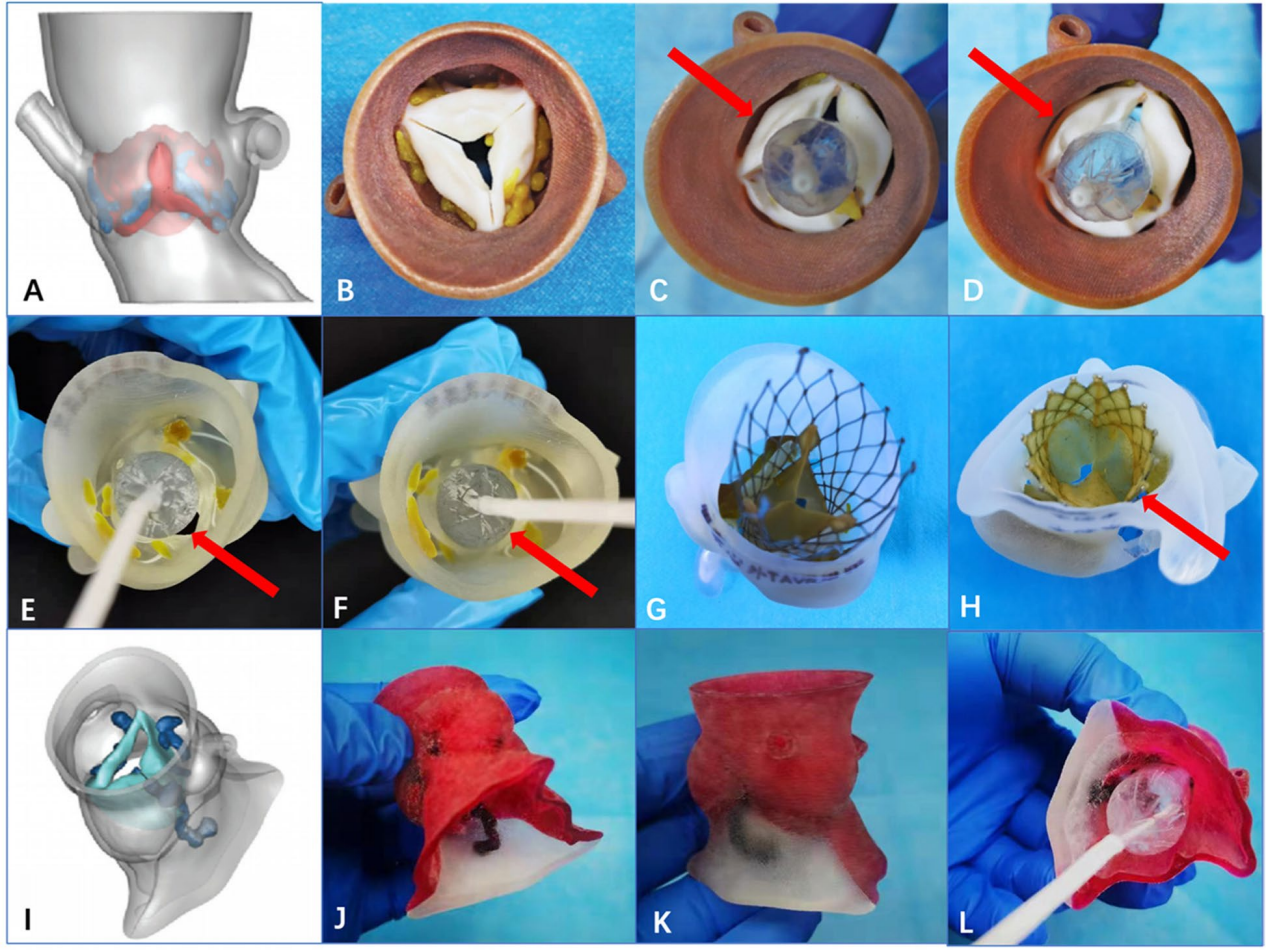
3-dimensional (3D) printed models were used to predict complications. **A** 3D reconstruction of the model. **B** The 3D printed model (ascending aorta view). **C, D** Predilation of the 3D printed model by using a balloon (Newmed Company, Shanghai, China) to observe the risk of coronary obstruction. Yellow arrows show that the area of the right coronary orifice was almost covered with different sizes of balloon dilations. **E-F** The 20-mm and 23-mm balloon (Newmed Company, Shanghai, China) was expanded in the model, respectively; red arrows show the position of PVL. **G-H** The release was simulated by using a Prizvalve® (Newmed Company, Shanghai, China) in the model (the ascending aorta view and the left ventricular outflow tract view); PVL could be seen (red arrow). **I** The left ventricular outflow tract (LVOT) and the calcification distribution could be observed in the 3D reconstructed models. **J-K** Multimaterial full-color 3D printing of the aortic root model (LVOT view and lateral view). **L** From the balloon (Newmed Company, Shanghai, China) dilation in vitro, leaflet displacement and interaction with the LVOT could be observed to predict of the incidence of conduction block. The white part of the model, which indicates the area between the anterior leaflet of the mitral valve and the membranous part of interventricular septum, is the possible section of conduction block

Heitkemper et al. (Heitkemper et al. 2020) and Young et al. (Young et al. 2020) showed an aortic root reconstructed by 3D printing preoperatively to analyze the distribution of calcification in order to avoid coronary obstruction. For high-risk patients, the 3D printed models of the aortic root can be used for the in vitro simulation of the incidence of coronary obstruction. Different sizes of balloons can be used to expand the aortic root to comprehensively evaluate the relationship between the leaflets, the sinus, and the coronary opening and the capacity of the sinus after dilation of the balloon to avoid the occurrence of coronary obstruction (Fig. 3A-D). At the same time, a demonstration stented valve may be used in the 3D printed model to observe the relationship between the stent and the coronary opening after the device is released.

### Paravalvular leakage

Paravalvular leakage is one of the most common complications after TAVR, and it directly affects the prognosis of the patients. The prediction of paravalvular leakage before surgery is important when planning the surgical strategy and selecting the prosthesis. Preprocedural evaluation of image data could identify the calcification in the leaflets and annulus but could not accurately determine the degree of paravalvular leakage. Qian et al. (Qian et al. 2017), Reiff et al. (Reiff et al. 2020) and Thorburn et al. (Thorburn et al. 2020) used a 3D printed model of the aortic root to predict the incidence of paravalvular leakage during TAVR and achieved good results. Meanwhile, researchers at Georgia Tech and the Piedmont Heart Institute used a multimaterial 3D printer to print models of the aortic root. Their model exhibited tissue physiology more clearly by controlling the diameter and bending the wavelength of the material. The model showed the specific structures of the leaflets, such as calcification and leaflet thickening (Maragiannis et al. 2014).

For patients with complex anatomical structures, the 3D printed model of the aortic root can be used for the in vitro simulation of the position and the size of the paravalvular leakage area (Fig. 3E-H). According to the simulation outcomes obtained when using the 3D printed model to plan the strategy for the TAVR, a relatively larger valve should be used so that the stent fits the annulus and the left ventricular outflow tract appropriately in case of reduced paravalvular leakage.

### Conduction block

Studies have shown that the incidence of cases of conduction block requiring a permanent pacemaker implant after TAVR is higher than that requiring surgical aortic valve replacement (Mahajan et al. 2021). Moreover, because the patients are relatively younger, researchers should pay more attention to conduction block after TAVR. Fujita et al. (Fujita et al. 2016) and Sammour et al. (Sammour et al. 2021) demonstrated that the cause of conduction block after surgery may be the displacement of calcification, which leads to continuous compression or permanent damage of the atrioventricular conduction system at the junction between the right coronary sinus and the noncoronary sinus. Studies have shown that an excessively low position of the stent increases the incidence of conduction block, especially left bundle branch block (Phan et al. 2020). Conduction block usually occurs immediately after the operation or up 24 to 48 hours later and often requires a permanent pacemaker implant (Aymond et al. 2021). Rocatello et al. (Rocatello et al. 2017) showed that 3D reconstruction of the aortic root has a clinically significant effect because it reduces the incidence of postprocedural conduction block. The 3D printed model of aortic stenosis associated with calcification can be used to implement balloon dilation and predict the direction of the migration. In addition, different stents were used to predict the incidence of conduction block: The authors simulated the position of the release in order to observe the influence of calcification on the position of release (Fig. 3I-L).

### Transcatheter aortic valve replacement simulation

**Fig. 4:**
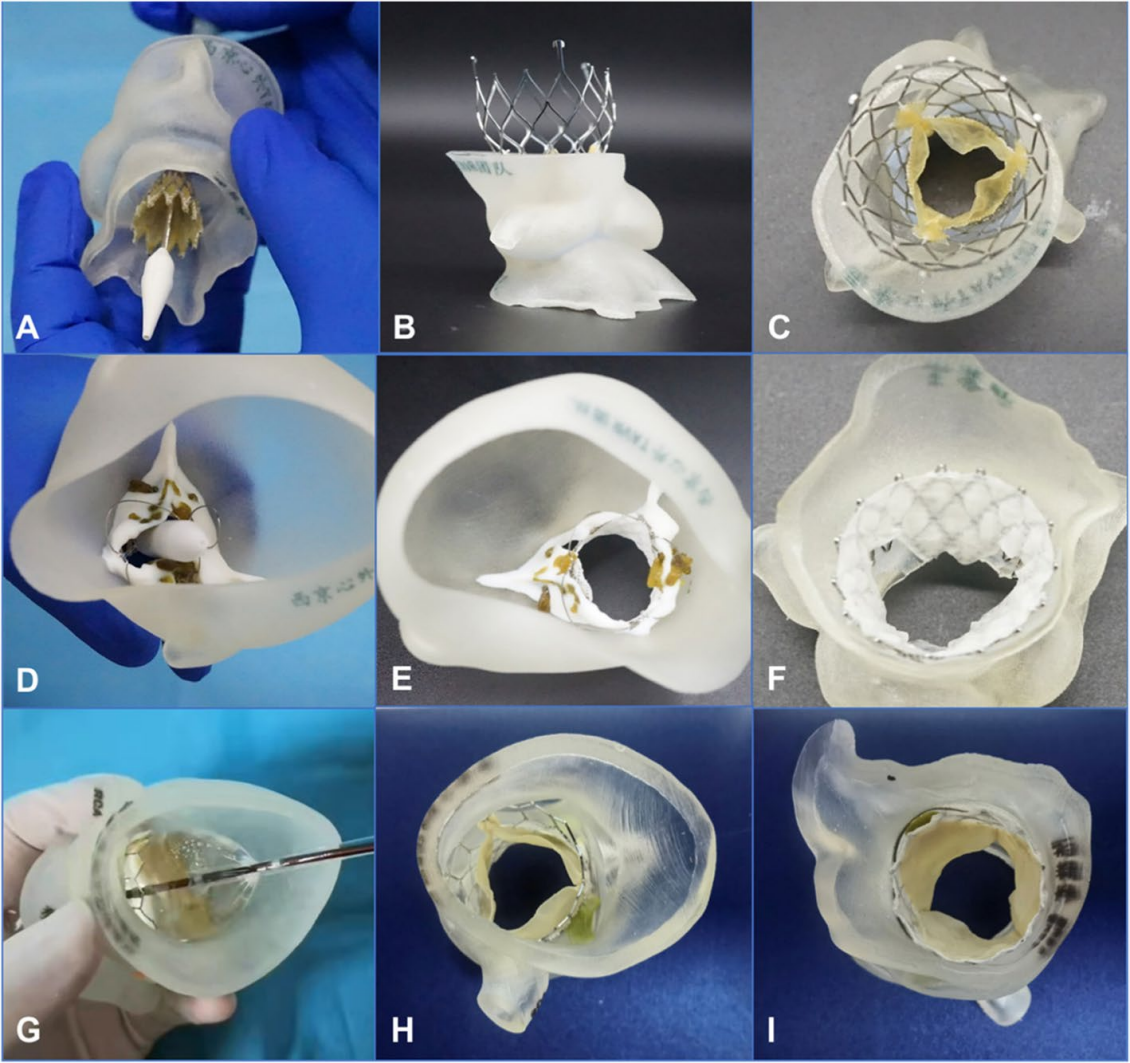
The release of different valves was simulated in the 3-dimensional printing models. **A-C** The simulated release of a Venus A valve (Qiming Company, Hangzhou, Jiangsu Province, China). **D-F** The simulated release of a J-valve (Jiecheng Company, Suzhou, Jiangsu Province, China). **G-I** The simulated release of a Prizvalve® (Newmed Company, Shanghai, China)

**Fig. 5:**
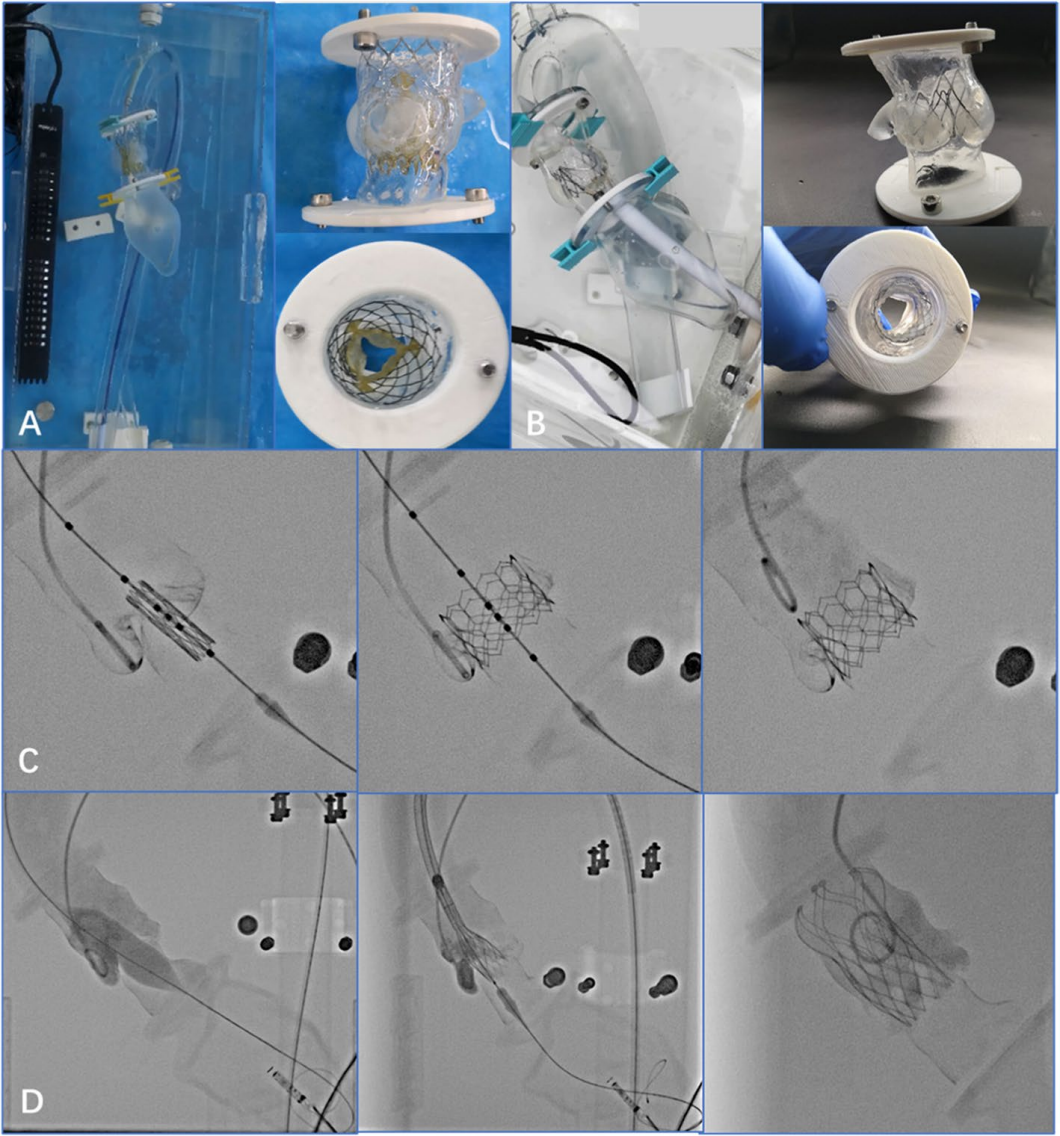
The simulated release of (TAVR) simulators. **A** Transfemoral TAVR simulator. **B** Transapical TAVR simulator. **C** The digital subtraction angiography images of the simulated release using a Prizvalve® (Newmed Company, Shanghai, China). **D** The digital subtraction angiography images of the simulated release using a VitaFlow valve (Microport, Shanghai, China)

The 3D printed aortic root model can provide effective preprocedural guidance for TAVR and further improve the accuracy of preprocedural evaluations (Vukicevic et al. 2017; Li et al. 2017). For some complex cases, preprocedural 3D printed models can be used for the simulation in vitro, which may be helpful to select prosthetic valves and determin the procedural results. With the guidance of the 3D printing simulator, doctors may confirm the position of the stent and the diameter of the annulus, thereby predicting the incidence of paravalvular leakage, conduction block, and other complications (Fig. 4). To better train young clinicians and to increase their proficiency during TAVR, a pulsatile platform is assembled to simulate procedures with individualized 3D printed aortic root models (Fig. 5). The platform may help young clinicians develop a clearer understanding of TAVR, shorten their learning curves, and avoid the occurrence of complications (Ma et al. 2021; Valverde 2017; Wang et al. 2018).

### Postprocedural evaluation

**Fig. 6:**
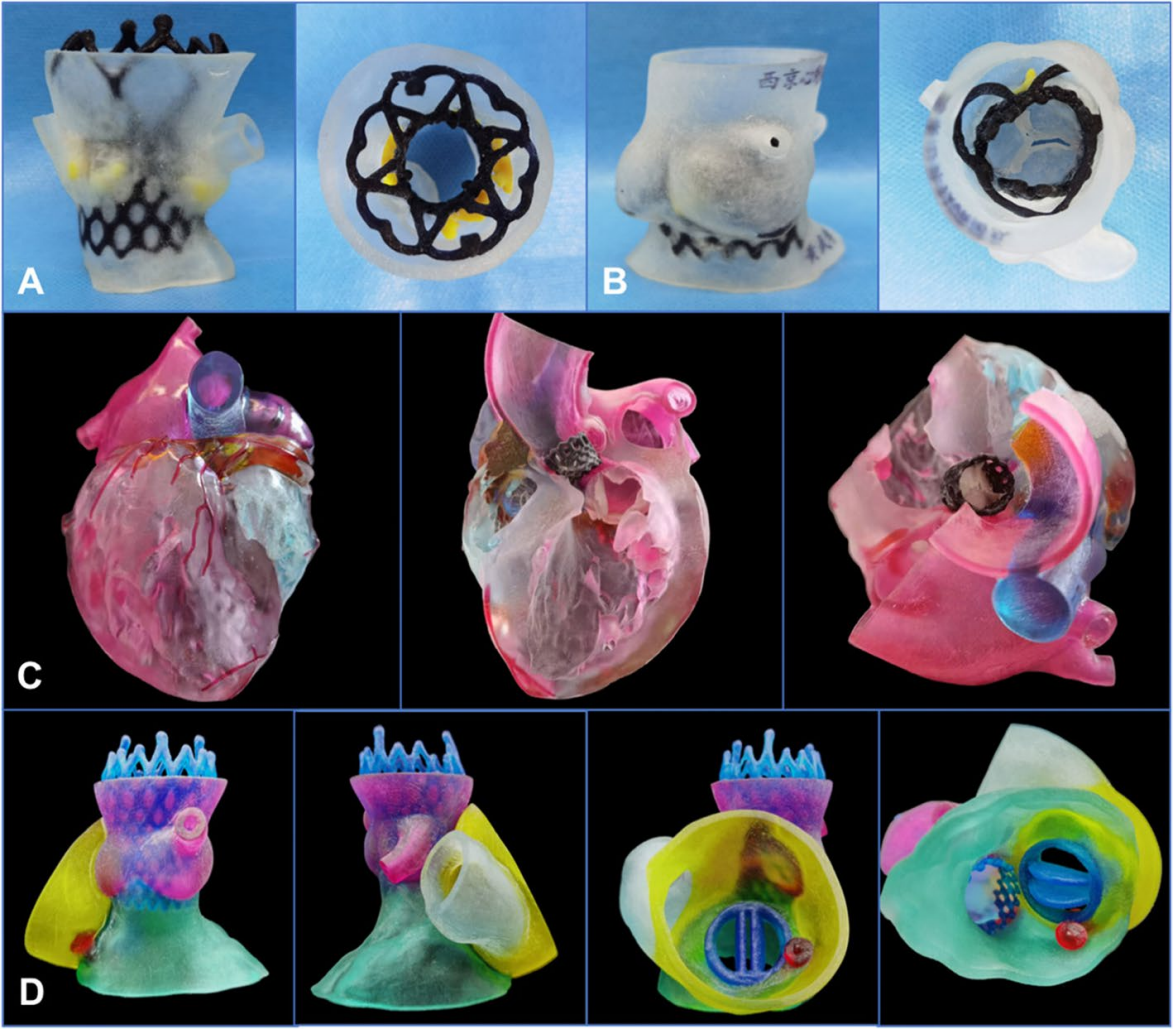
The postprocedural evaluation after transcatheter aortic valve replacement using 3-dimensional printing models. **A** The postprocedural model used a VitaFlow valve (Microport, Shanghai, China) (the lateral view and the ascending aorta view). **B** The postprocedural model used a J-valve (Jiecheng Company, Suzhou, Jiangsu Province, China) (the positive view and the ascending aorta view). **C** The postprocedural multicolor model used a Prizvalve® (Newmed Company, Shanghai, China) (profile view and ascending aorta view). **D** Different views of the 3-dimensional printed model after transcatheter aortic valve replacement combined with mechanical mitral valve replacement and paravalvular leakage occlusion

Echocardiography is often used to observe the position of the prosthesis, the location of the stent, and the degree of paravalvular leakage when evaluating the postprocedural results of TAVR (Naqvi 2018; Welle et al. 2021). Postprocedural CT data can be used to measure the depth of the stent, the coaxiality, and size of the annulus. However, image data may not directly show the stent in the aortic root or the relationship between the leaflets and the coronary sinus. As a result, the 3D reconstructed printed model of the aortic root helps surgeons to observe the anatomical structures clearly (Fig. 6).

## The future directions of 3-dimensional printing in transcatheter aortic valve replacement

In recent decades, the rapid development of TAVR worldwide has provided new methods for minimally invasive treatment of structural heart diseases. More and more older patients with severe valvular disease have been treated effectively (Zhou et al. 2018). The 3D printed models of the aortic root can help medical students and surgeons have a better understanding of the anatomical structures. With the simulation of balloon dilation and stent implantation, various TAVR-related complications may be effectively avoided. The pulsatile 3D printed simulation platform can effectively simulate the human hemodynamic environment to provide a realistic in vitro platform for product testing and development and to effectively improve the development of related surgical devices. In addition, using 3D printed models can also improve the learning curve of surgeons and young doctors. Along with the development of TAVR, 3D printing has been widely applied in device testing, in vitro simulation, and other areas (Vukicevic et al. 2017; Garner and Singla 2019). Interventional treatments of structural heart diseases have been carried out worldwide. We believe that cardiovascular 3D printing will play an important role in guiding interventional treatments, which may effectively reduce the operating time and amount of exposure to intraprocedural digital subtraction angiography (Yang 2020; Ripley et al. 2016).

The author’s team used 3D printing to assist in guiding TAVR and completed 682 cases of TAVR from January 2018 to March 2022, with significant reduction in postprocedural complications, models can help clinicians make the diagnosis and procedural planning more accurate, and reduce X-ray exposure time and blood loss. However, there are still the following limitations: Higher resolution research and post-processing of the images require additional time and expense, and expertise and cost may be an issue for many medical centers. In addition, current printing materials do not adequately reflect tissue physiology; thus device-tissue interactions are a challenge that needs to be solved. With the limitation above, there are some potential solutions to solve the problems. Firstly, further researches will be developed in the materials and equipment of cardiovascular 3D printing; Secondly, now there are researchers focusing on the development of soft tissue materials, and it is believed that the materials will be upgraded in the next five years; Thirdly, the curative effect of TAVR will continue to improve as the balloon-expanded valve becomes more widely used.

Currently, 3D printing can be used to reconstruct the anatomical structure of the aortic root and provide corresponding guidance to avoid complications during TAVR and can support teaching and training by simulating operations. However, it is not possible thus far to simulate implantable instruments like those used in orthopedics and stomatology. However, we believe that with the continuous development of materials and bioprinters, precise bioprinting will eventually be able to duplicate individual anatomical structures, and implantable organs such as bioprinted blood vessels, valves, and even the whole heart will also be gradually realized (Gilbert et al. 2018; Daly et al. 2021).

## Conclusion

In this review, 3D printed models of aortic root have been displayed which may be functioned in the preprocedural assessment and postprocedural evaluation. Overall, cardiovascular 3D printing plays an important guiding role in the perioperative treatments of TAVR, and of course, this innovative technology will have a broad prospect in the future.

## Data Availability

All the 3D printed models and simulators which were used in this review were supplied by Make Medical Technology Co., LTD. (Xi’an, China).

## Abbreviations

TAVR: Transcatheter Aortic Valve Replacement
3D: 3-Dimensional
CT: Computed Tomography
DICOM: Digital Imaging and Communications in Medicine
STL: Standard Tessellation Language
